# Electrocorticographic Changes and Neuronal Maturation in the Antidepressant-like and Anxiolytic Effects of Micro- or Macrodosing of *Psilocybe cubensis* Mushroom in Mice †

**DOI:** 10.3390/molecules31081331

**Published:** 2026-04-18

**Authors:** Flor Eréndira Sánchez-Cortés, Nelly Maritza Vega-Rivera, Raúl Escamilla-Orozco, David Martínez-Vargas, Alberto Hernandez-Leon, Ingrid Escamilla-Cervantes, Aylin R. Tabal-Robles, Martín Torres-Valencia, Leticia Romero-Bautista, María Eva González-Trujano, Erika Estrada-Camarena

**Affiliations:** 1 Laboratorio de Neurofarmacología de Productos Naturales, Dirección de Investigaciones Biomédicas en Salud Mental, Instituto Nacional de Psiquiatría Ramón de la Fuente Muñiz (INPRFM), Calz. México-Xochimilco 101, Col. San Lorenzo-Huipulco, Tlalpan, Ciudad de México 14370, Mexico; fesctdl@gmail.com (F.E.S.-C.); albertoh-leon@hotmail.com (A.H.-L.); ingridcer.49@gmail.com (I.E.-C.); 2 Departamento de Neurociencias, Facultad de Medicina, Universidad Nacional Autónoma de México, Copilco Universidad, Ciudad Universitaria, Ciudad de México 04360, Mexico; 3 Laboratorio de Neuropsicofarmacología, Dirección de Investigaciones Biomédicas en Salud Mental, Instituto Nacional de Psiquiatría Ramón de la Fuente Muñiz, Calz. México-Xochimilco 101, Col. San Lorenzo-Huipulco, Tlalpan, Ciudad de México 14370, Mexico; vegquim2909@gmail.com; 4 Servicios Clínicos, Dirección de Servicios Clínicos, Instituto Nacional de Psiquiatría Ramón de la Fuente Muñiz, Calz. México-Xochimilco 101, Col. San Lorenzo-Huipulco, Tlalpan, Ciudad de México 14370, Mexico; rescam01@gmail.com; 5 Laboratorio de Neurofisiología del Control y la Regulación, Dirección de Investigaciones Biomédicas en Salud Mental, Instituto Nacional de Psiquiatría Ramón de la Fuente Muñiz, Calz. México-Xochimilco 101, Col. San Lorenzo-Huipulco, Tlalpan, Ciudad de México 14370, Mexico; davmv2@gmail.com; 6 Área Académica de Química, Universidad Autónoma del Estado de Hidalgo, Km. 4.5 Carretera Pachuca-Tulancingo, Mineral de la Reforma, Hidalgo 42184, Mexico; ta260266@uaeh.edu.mx (A.R.T.-R.); martin@uaeh.edu.mx (M.T.-V.); 7 Laboratorio de Micología Integral, Área Académica de Biología, Universidad Autónoma del Estado de Hidalgo, Km 4.5 Carretera Pachuca-Tulancingo, Mineral de la Reforma, Hidalgo 42184, Mexico; romerob@uaeh.edu.mx

**Keywords:** anxiety, dendritic maturation, depression, electrocorticogram, macrodose, microdose, *P. cubensis* mushroom

## Abstract

Mushroom use dates back to ancient times, and it currently remains significant among indigenous and urban populations as a medicinal option. *Psilocybe* species are suggested to modify emotions when administered in macro- or microdose form for the treatment of anxiety and depression, both often affected by a delayed onset and adverse effects of current pharmacological therapy. The objective of this study was to evaluate the anxiolytic and/or antidepressant-like effects of *P. cubensis* mushroom aqueous extract (*Pc*AE) microdosing in mice using open-field and rota-rod tests, followed by plus-maze or forced swimming tests. We also evaluated changes in neuronal activity and dendritic maturation using electrocorticography (ECoG) and immunohistochemical techniques. The outcomes were compared with an effective macrodose of *Pc*AE and antidepressant fluoxetine (FLX). For this study, mice were grouped as follows: (1) vehicle, (2) acute, and (3) repeated (10 days) *Pc*AE microdosing (1 µg/kg); (4) single *Pc*AE macrodose (1 g/kg); and (5) acute and (6) repeated reference drug fluoxetine (FLX, 10 mg/kg).The anxiolytic and antidepressant-like effects using microdosing were similar to those observed with macrodoses of *Pc*AE and FLX; significant dose- and/or time-dependent changes in the ECoG and dendritic maturation of hippocampus neurons were also observed, in addition to altered corticosterone levels. To conclude, *P. cubensis* mushroom promotes brain effects in mice after micro- and macrodosing, supporting its potential as a therapeutic alternative for mental health.

## 1. Introduction

The medical knowledge of various cultures, such as the Mesoamerican–pre-Columbian cultures and others, has left an important heritage for the development of universal medicine. The representation of illnesses in pre-Columbian ceramic figures allowed identifying typical characteristics that could be considered representative of a depressive state, suggesting that psychiatric disorders, such as depression, were not only present in pre-Hispanic Mexico but also identified before the arrival of the Spanish on the American continent [1,2]. Currently, major depressive disorder (MDD) is a disease characterized by an emotional state of desperateness; demotivation; unhappiness; loss of interest or ability to enjoy things that used to bring pleasure; feelings of guilt; feelings of insignificance; and, in several cases, suicidal tendencies or thoughts of death, denoting a major public health problem [3,4]. The World Health Organization (WHO) has projected that 3.8% of the population in the world may experience depression, with depression being one of the greatest public mental disorders that can occur in isolation or in comorbidity with other conditions, such as anxiety or other central nervous system (CNS) syndromes [5]. The peak prevalence of MDD occurs during adolescence and early adulthood [6], and its recognition has been increased recently by the SARS-CoV-2 pandemic [7]. When anxiety symptomatology appears during preadolescence, it can lead to the development of depression [8]. Pharmacological management consists of selective serotonin and/or norepinephrine reuptake inhibitors (SSRIs/SNSIs) or tricyclics, which have been associated with adverse effects. In addition, it can take up to 6 weeks to achieve beneficial effects. One of the most widely reported treatments may be fluoxetine (FLX) due to its efficacy; cost; and, most importantly, tolerability, which depends on the patient’s response [9]. However, the lack of rapid efficacy of antidepressants and the presence of common adverse effects—alone or related to external reasons (such as socioeconomic factors)—may lead to poor adherence or discontinuation of treatment, resulting in relapses and worsening the quality of life of patients [10,11].

The variable nature of MDD across individuals, along with the factors involved in traditional treatment, indicates the need to seek alternative therapies with greater efficacy, faster onset than standard treatments, and fewer adverse effects. Such is the case with the limited impact of conventional antidepressants on treatment-resistant major depressive disorder (TRD), described as a depressive disorder that does not achieve sufficient remission after treatment, where dose increases sometimes fail to address tolerability issues due to interindividual variability. A viable option for new treatments for this disorder is the search for compounds with different mechanisms of action. Psilocybin and psilocin are globally distributed tryptophan-indole-based alkaloids found in *Psilocybe* mushrooms. They are currently being extensively studied in clinical and some preclinical studies to provide evidence of their potential efficacy not only as an antidepressant but also as an anxiolytic alternative [12,13,14]. According to anecdotal reports and observational studies, symptoms of stress, anxiety, and depression may also be improved using microdosing, suggesting that this practice may improve mood and cognitive function but also pointing to a possible physiological mechanism [15].

Microdosing typically involves a sub-hallucinogenic dose of a psychedelic compound [16]. Few studies have investigated the antidepressant and anxiolytic properties of microdosing in humans; thus, any therapeutic effect may be associated with a placebo effect. As for studies in animal models, which could reduce the bias caused by expectations, they are still scarce. Therefore, further research is needed on the therapeutic effects of not only psilocybin or psilocin but also the entire mushroom to provide more confidence and evidence about the therapeutic potential and safe use of microdosing and/or macrodosing. Recent studies have reported that psilocybin at macrodoses can promote neurogenesis and spinogenesis in mouse models [17]. In human neurons, psilocybin also promoted structural changes by increasing synaptic density (synaptogenesis) [18]. However, there are not enough studies in the literature on the neuronal effects of macro-and/or microdosing *Psilocybe* mushrooms. In a preliminary report, our group reported a significant and dose-dependent antidepressant-like effect of an aqueous extract of *P. cubensis* (*Pc*AE) across doses ranging from 1 to 100 mg/kg (i.p.) and even at 1000 mg/kg, p.o. [14]. ECoG at 100 mg/kg, i.p., or 1000 mg/kg, p.o, exhibited CNS depressant activity without paroxysmal-like activity or abnormal electrophysiological patterns, such as burst suppression patterns detectable by ECoG in the cerebral frontal cortex of mice [14]. On the other hand, to our knowledge, neuroplasticity studies for *Psilocybe* mushrooms have not been explored at all.

Mexico is a country with the greatest quantity of *Psilocybe* mushroom species [19] recorded and described as Teonanacatl due to its narcotic and medicinal uses among the indigenous Mexican people during the period of the Spanish conquest [20]. Furthermore, Mexico represents, without a doubt, the richest area in the world in terms of diversity and use of hallucinogens/entheogens by indigenous cultures [21]. It is a region with 10% of the world’s total biodiversity. Archaeological and historical evidence of the use of *Psilocybe* mushrooms by Mesoamerican people 3000 years ago describes their utilization in rituals and therapies related to the CNS [2,21]. The civilizations that flourished in Mesoamerica were diverse, displaying their great experience and ability in the use of abundant hallucinogens. The purpose of using these compounds was to achieve greater open-mindedness [21]. At present, several indigenous people are still using *Psilocybe*, such as the Nahuas and Matlazincs in Central Mexico. In Oaxaca, the Mazatecs, Zapotecs, Mixes, Mixtecs, Chinantecs, and Chatinos still use these mushrooms, and Mayan, Purepechas, and Otomies peoples, as well as indigenous peoples near the Nevado de Colima volcano, have demonstrated ritual and medicinal uses that have been practiced for centuries, even millennia, in Mexico [22,23].

To date, few experimental scientific reports have investigated the effects of complete *P. cubensis* mushroom dosing on the CNS, and our group aims to continue the search for evidence to support the therapeutic uses of this natural product through a wide range of doses. In this study, the pharmacological effects of *P. cubensis* mushrooms after an acute or repeated administration of microdosing were investigated to support their anxiolytic and/or antidepressant behavioral responses. We explore neuronal in situ activity by carrying out ECoG recordings in the brain parietal cortex of mice, and the possible neuroplasticity changes after immunohistochemical analyses of dendritic maturity are explored in the dentate gyrus of the hippocampus region; the outcomes were compared with respect to a macrodose of the *P. cubensis* mushroom and FLX, a reference drug used in clinical therapy.

## 2. Results

### 2.1. Presence of Psilocybin in the PcAE

**Figure 1:**
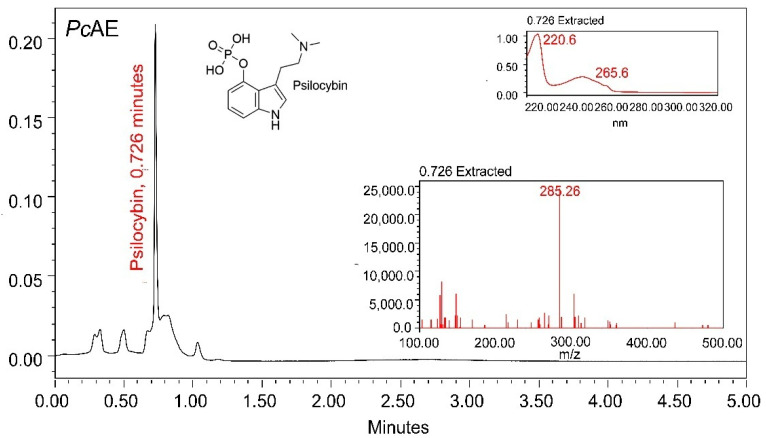
UPLC-MS chromatographic profile of *Pc*AE reflecting the presence of the bioactive metabolite psilocybin.

Previous chemical analyses of *Psilocybe* mushrooms have reported the existence of different constituents other than alkaloids, including amino acids, sesquiterpenes, sterols, and carbohydrates. However, most chemical studies on these mushrooms have been focused on the identification, quantification, and/or isolation of psilocybin and psilocin. To verify the presence of psilocybin in a *P. cubensis* aqueous extract (*Pc*AE), a UPLC-MS analysis was carried out to confirm the presence of this alkaloid in the *Pc*AE, characterized according to a retention time of 0.726 min, and the UV-MS spectra are shown in Figure 1. The mass spectrum of psilocybin, as a “fingerprint” of the molecule, is included as a reference, fragmenting it and measuring the mass-to-charge ratio, as it is considered a biomarker of the genus *Psilocybe.* However, further fractionation, isolation, and purification of this alkaloid and other metabolites will be required to characterize the bioactive compounds responsible for the activity of *P. cubensis*.

### 2.2. ECoG Analysis After an Acute or Repeated Microdosing of PcAE

**Figure 2:**
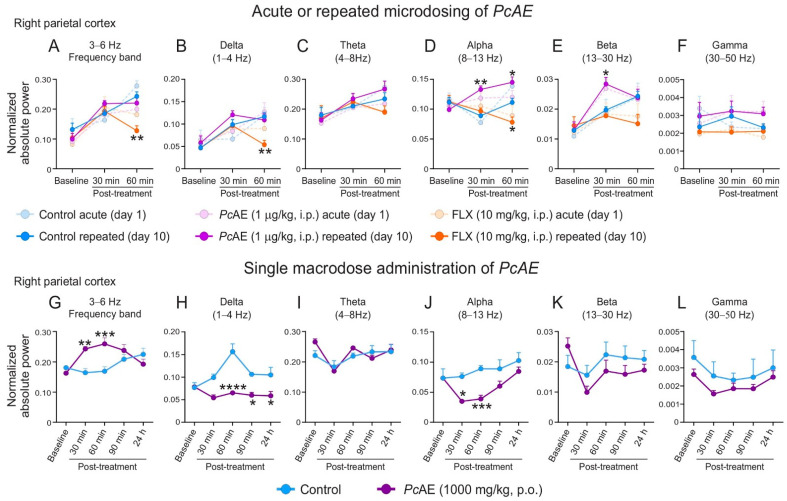
Normalized absolute power of electrocorticographic activity from the right parietal cortex of treated mice at baseline and at 30 and 60 min after acute or repeated administration of *P. cubensis* aqueous extracts (*Pc*AE, 1 µg/kg, i.p.) or fluoxetine (10 mg/kg, i.p.) (**A**–**F**) and at baseline, 30, 60, and 90 min, and 22 h after a single macrodose administration of *Pc*AE (1000 mg/kg, p.o.) (**G**–**L**). Data are presented as mean ± standard error of the mean (S.E.M.) from six repetitions. Two-way repeated-measure ANOVA followed by Tukey’s or Sidak’s post hoc test (for multiple or pairwise comparisons, respectively) was applied for the microdosing or macrodose treatment. * *p* < 0.05, ** *p* < 0.01, *** *p* < 0.001, and **** *p* < 0.0001 vs. control group.

The spectral power analyses of the absolute power in the right parietal cortex exposed statistical interactions between factors of time and treatment, indicating that the treatment’s effect on the frequency bands varies over time (Figure 2). Ten consecutive days of FLX administration (10 mg/kg, i.p.), as the reference drug, caused a significant decrease in the 3–6 Hz frequency range (treatment F_2,15_ = 4.84, *p* = 0.0238; time F_2,30_ = 21.50, *p* < 0.0001; interaction F_4,30_ = 3.67, *p* = 0.0150; Figure 2A); delta band (treatment F_2,15_ = 4.61, *p* = 0.0275; time F_2,30_ = 21.68, *p* < 0.0001; interaction F_4,30_ = 2.87, *p* = 0.0398; Figure 2B); and alpha band (treatment F_2,15_ = 18.39, *p* < 0.0001; time F_2,30_ = 0.22, *p* = 0.7977; interaction F_4,30_ = 5.30, *p* = 0.0024) at 60 min post-treatment vs. the control group. A decreasing trend was detected in the beta band (Figure 2E). In the acute or repeated microdosing of *Pc*AE, a similar response profile was observed between these treatments in cortical activity, mainly for repeated administrations, as an increase in the alpha (treatment F_2,15_ = 18.39, *p* < 0.0001; time F_2,30_ = 0.22, *p* = 0.7977; interaction F_4,30_ = 5.30, *p* = 0.0024; Figure 2D) and beta (treatment: F_2,15_ = 6.27, *p* = 0.0105; time F_2,30_ = 12.09, *p* = 0.0001; interaction F_4,30_ = 3.05, *p* = 0.0319; Figure 2E) bands was observed at 30 min and 60 min, or only at 30 min, respectively. Theta and gamma bands did not exhibit differences among the treatments with FLX or *Pc*AE or acute or repeated administration vs. the control group (Figure 2C and Figure 2F, respectively).

### 2.3. ECoG Analysis of a PcAE Macrodose

The ECoG response in the single-microdose *Pc*AE administration was primarily associated with an enhancement of oscillatory activity in the 3–6 Hz frequency band (Figure 2G). A marked increase in normalized absolute power was observed in this band at 30 and 60 min vs. the control group (treatment F_1,10_ = 77.32, *p* < 0.0001; time F_4,40_ = 0.9584, *p* = 0.4408; interaction F_4,40_ = 6.23, *p* = 0.0005; Figure 2G). This increase was accompanied by a decrease in the delta band (treatment F_1,10_ = 52.15, *p* < 0.0001; time F_4,40_ = 3.54, *p* = 0.0144; interaction F_4,40_ = 4.37, *p* = 0.0050; Figure 2H) at 60 min, 90 min, and 24 h with respect to the control group. Additionally, a decrease in the alpha band (treatment F_1,10_ = 9.83, *p* = 0.0106; time: F_4,40_ = 7.48, *p* = 0.0009; interaction F_4,40_ = 2.30, *p* = 0.0753; Figure 2J) was observed at 30 and 60 min, compared to the control group. The theta, beta, and gamma bands were not modified in the presence of *Pc*AE (Figure 2I and Figure 2L, respectively).

### 2.4. Behavioral Responses

#### 2.4.1. Anxiolytic Activity of Acute and Repeated Microdosing of *PcAE*

**Figure 3:**
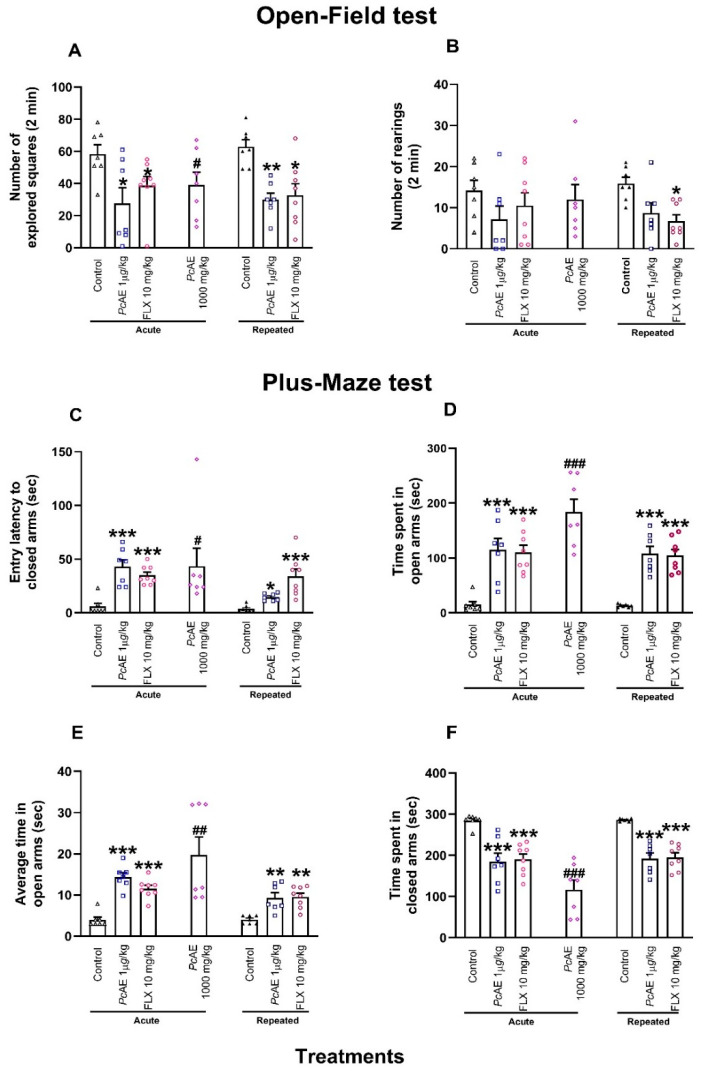
Pharmacological effects of acute macrodosing and microdosing of an aqueous extract of *P. cubensis* mushroom (*Pc*AE, 1000 mg/kg and 1 µg/kg, i.p.) and fluoxetine (FLX10, 10 mg/kg, s.c., 3 doses) after repeated administration for 10 days (*Pc*AE, 1 µg/kg, or FLX10, 10 mg/kg, i.p.) in comparison to the control in an open-field test carried out on mice; the following were registered: (**A**) number of squares and (**B**) number of rearings. Plus-maze test describing the (**C**) latency to first entry, (**D**) time spent in open arms, (**E**) average time in open arms, and (**F**) time spent in closed arms observed within 5 min. Data are shown as mean ± S.E.M. of at least six animals. Two-way ANOVA followed by post hoc Tukey’s test, and * *p* < 0.05, ** *p* < 0.01, and *** *p* < 0.001 vs. the acute or repeated control group. An unpaired Student’s t-test was used to compare the acute *Pc*AE macrodose (1000 mg/kg) vs. the acute control: # *p* < 0.05, ## *p* < 0.01, and ### *p* < 0.001.

Mice receiving acute or repeated microdosing of *Pc*AE (1 µg/kg, i.p.) exhibited a significant reduction in the number of squares crossed in the OFT compared to the control group. A similar response was observed after the administration of FLX10 (10 mg/kg, s.c.) (treatment F_2,38_ = 12.93, *p* < 0.0001; time F_1,38_ = 0.005, *p* = 0.9400; interaction F_2,38_ = 0.37, *p* = 0.6800) (Figure 3A). Mice receiving macrodoses also exhibited significant differences vs. the control mice (t = 2.21, df = 12, *p* = 0.0460) (Figure 3A). In contrast, rearing behavior in the OFT was only significatively reduced in mice receiving repeated administrations of FLX10 (Figure 3B).

Regarding the behavioral evaluation of mice in the elevated plus maze test (EPM), the latency to enter the closed arms significantly increased in the presence of acute and repeated administrations of *Pc*AE microdoses or reference drug FLX10 (treatment F_2,38_ = 26.04, *p* < 0.0001; time F_1,38_ = 8.76, *p* = 0.0050, interaction F_2,38_ = 6.15, *p* = 0.0040). The effect of microdosing *Pc*AE was more important after acute than repeated administrations vs. the control group (Figure 3C). In a similar manner, acute macrodosing significantly enhanced this response (t = 2.21, df = 12, *p* = 0.0474) (Figure 3C).

The time spent by mice in the open arms was also increased following both acute or repeated administration of *Pc*AE microdosing or FLX10 (treatment F_2,38_ = 40.72, *p* < 0.0001; time F_1,38_ = 9.74, *p* = 0.003; interaction F_2,38_ = 2.38, *p* = 0.03), as well as with *Pc*AE macrodoses (t = 7.00, *p* < 0.0001) (Figure 3D). Similarly, average permanence in the open arms (treatment F_2,38_ = 42.91, *p* < 0.0001; time F_1,38_ = 10.23, *p* = 0.002; interaction F_2,38_ = 3.92, *p* = 0.028), and in *Pc*AE macrodoses (t = 3.57, df = 12, *p* = 0.0039) (Figure 3E) increased. A complementary decrease in permanence for mice in the closed arms was detected in both single and repeated treatments of *Pc*AE or FLX10 microdosing (treatment F_2,38_ = 37.60, *p* < 0.0001; time F_1,38_ = 0.23, *p* = 0.63; interaction F_2,38_ = 0.022, *p* = 0.97), as well as in the presence of acute *Pc*AE macrodoses (t = 6.98, df = 12, *p* < 0.0001) (Figure 3F).

#### 2.4.2. Antidepressant-like Activity After Acute and Repeated Microdosing of *PcAE*

**Figure 4:**
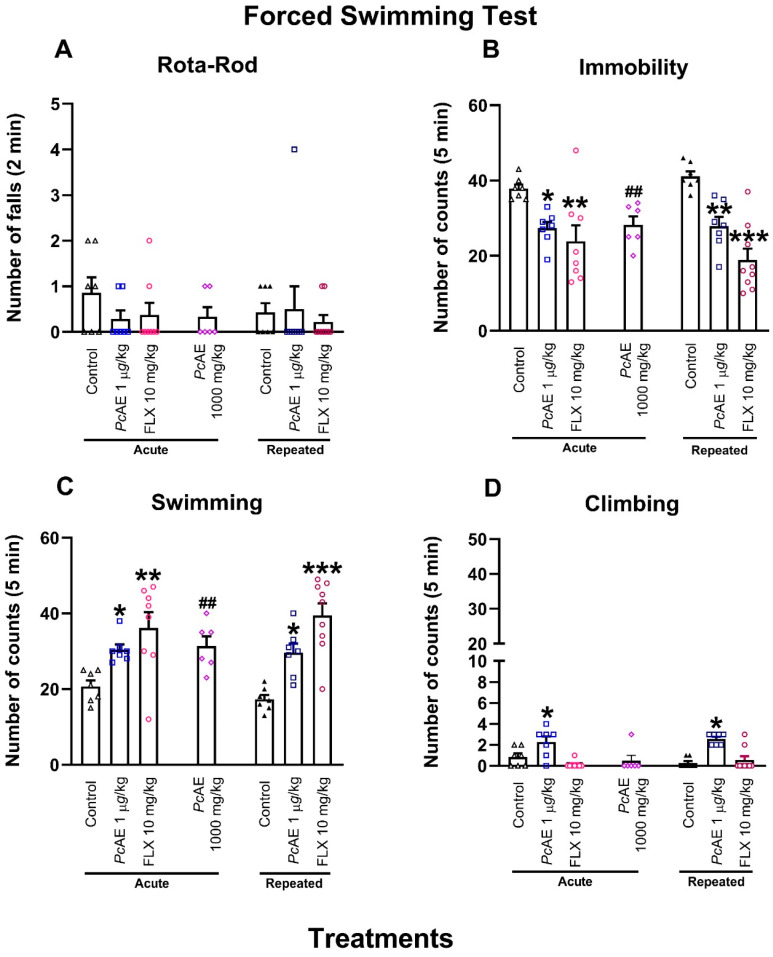
Pharmacological effects of acute and repeated microdosing of an aqueous extract of *P. cubensis* mushroom (*Pc*AE, 1 µg/kg, i.p.), single macrodose (1000 mg/kg), and the reference drug fluoxetine (FLX10, 10 mg/kg) compared to the vehicle group (control) in the rota-rod and forced swimming tests carried out on mice. (**A**) Rota-rod, (**B**) immobility, (**C**) swimming, and (**D**) climbing behaviors in mice. Data are shown as mean ± S.E.M. of at least six repetitions. Two-way ANOVA followed by post hoc Tukey’s test; * *p* < 0.05, ** *p* < 0.01, and *** *p* < 0.001 vs. the acute or repeated control group. An unpaired Student’s *t*-test was used to compare the acute *Pc*AE macrodose (1000 mg/kg) vs. the acute control; ## *p* < 0.01.

First, a rota-rod test was assessed to observe whether treatments modify motor coordination and could affect mouse performance in FST. No significant change in rod activity was observed in mice receiving FLX10 and acute or repeated administrations of *Pc*AE microdosing compared to the vehicle group or after an acute macrodose of *Pc*AE (Figure 4A) as observed in a preliminary evaluation [14].

With respect to immobility behavior, *Pc*AE microdosing promoted a significant reduction after its acute or repeated administration in the FST, similarly to the phenomenon observed in mice treated with the antidepressant reference drug FLX10 compared to the control (treatment F_2,39_ = 23.59, *p* < 0.0001; time F_1,39_ = 0.0290, *p* = 0.8600; interaction F_2,39_ = 1.28, *p* = 0.2800) (Figure 4B). This effect was also significant in the presence of acute macrodoses of *Pc*AE (t = 2.85, df = 12, *p* = 0.0145), as observed in a preliminary evaluation [14].

In a complementary manner, the swimming behavior of mice was increased in the presence of acute or repeated *Pc*AE microdosing, as well as in the FLX group vs. control mice (treatment F_2,39_ = 24.03, *p* < 0.0001; time F_1,39_ = 0.02, *p* = 0.8800; interaction F_2,39_ = 0.80, *p* = 0.4500) (Figure 4C). It is important to mention that the antidepressant-like activity of *Pc*AE microdosing after acute or repeated administration remained at almost the same levels observed in the preliminary macrodosing results (t = 2.43, df = 12, *p* = 0.0316) [14].

On the other hand, the climbing behavior of mice was changed in the presence of acute or repeated *Pc*AE microdosing compared to the vehicle group (treatment F_2,39_ = 24.49, *p* < 0.0001; time F_1,39_ = 0.03, *p* = 0.8500; interaction F_2,39_ = 1.37, *p* = 0.2600) but not after acute or repeated administrations of FLX10 or acute macrodosing treatments of *Pc*AE (Figure 4D).

### 2.5. Effect of Microdosing or Macrodosing of PcAE on Immunoreactive Doublecortin-Positive Cells

**Figure 5:**
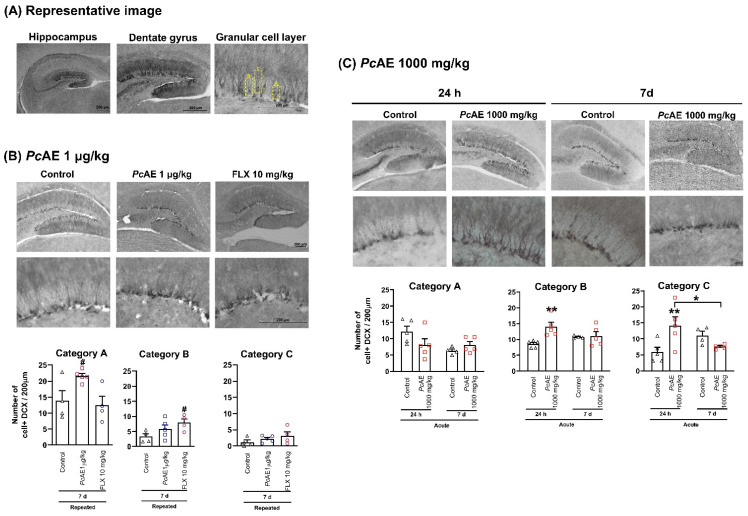
(**A**) Representative images of the morphological classification of granular cells identified along the 200 µm dentate gyrus area of the hippocampus marked with doublecortin (DCX). (**B**) Number of cells counted in mice receiving vehicle (control), aqueous extract microdose of *P. cubensis* mushroom (*Pc*AE, 1 µg/kg, i.p.), and the reference drug FLX (FLX10, 10 mg/kg, 3 doses) after repeated administration for 10 days. Data are shown as mean ± S.E.M. An unpaired Student’s *t*-test was used to compare the repeated treatment vs. the control group, # *p* < 0.05, *n* = 4–5 repetitions. (**C**) Number of cells counted from mice receiving a macrodose of *Pc*AE. Data are shown as mean ± S.E.M. Two-way ANOVA followed by Tukey’s test, * *p* < 0.05 and ** *p* < 0.01, *n* = 4–5 repetitions.

As no substantial difference was observed in electrical activity after ECoG analysis (Figure 2) or in the anxiolytic- or antidepressant-like effects, between acute and repeated administrations of *Pc*AE (Figure 3 and Figure 4, respectively), the immunohistochemical analysis for dendritic maturation was carried out only after repeated microdosing treatment (daily for 10 days) or FLX10 and after 7 days of the last administration (Figure 5). These results were compared to the acute macrodose treatment of *Pc*AE, which was evaluated both 24 h after administration and 7 days after the last dose (Figure 5).

Dendritic morphological changes were observed in the hippocampus along the 200 µm dentate gyrus area by observing granular cell layers identified as categories A to C (Figure 5). The DCX-positive cells of category A comprised the majority after repeated *Pc*AE microdosing administration (t = 2.62, df = 7, *p* = 0.0343), with a tendency to increase category B cells by producing a duplicate number vs. the control group; in contrast, FLX reached significance in this category of cells due to a more homogeneous response (t = 2.87, df = 6, *p* = 0.0285). Finally, in category C, both *Pc*AE and FLX did not exhibit changes (Figure 5B).

Regarding dendritic morphology in the case of the acute macrodosing of *Pc*AE, significant and marked DCX-positive cells enhancement was observed compared to repeated microdosing (Figure 5C). This effect was observed early during 24 h post-treatment with respect to DCX-labelled category B (treatment F_1,15_ = 7.07, *p* = 0.0179; time F_1,15_ = 0.095, *p* = 0.7615; interaction F_1,15_ = 5.58, *p* = 0.0320) and C (treatment F_1,15_ = 1.76, *p* = 0.0204; time F_1,15_ = 0.16, *p* = 0.6980; interaction F_1,15_ = 10.97, *p* = 0.0050) cells. Significant changes disappeared 7 days after treatment, as the values resembled those observed in the control group (Figure 5C).

**Figure 6:**
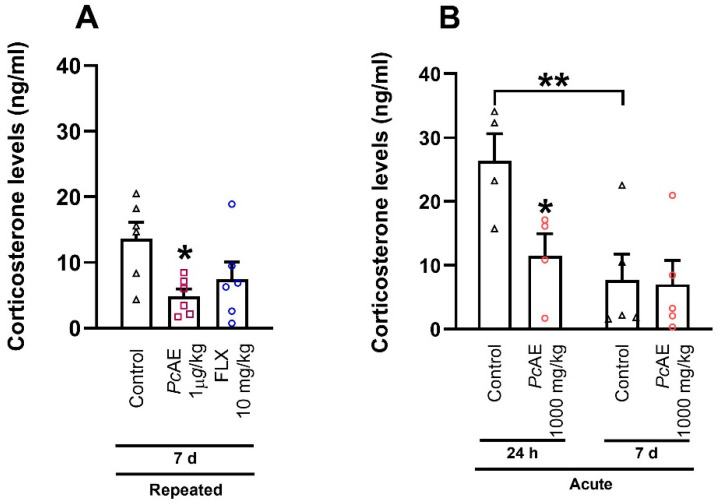
Effects on corticosterone levels at 7 days and/or 24 h after last administration of (**A**) repeated microdosing of an aqueous extract of *P. cubensis* mushroom (*Pc*AE) and the reference drug fluoxetine (FLX10, 10 mg/kg, i.p.) (treatment for 10 days); or after (**B**) an acute macrodose of *Pc*AE compared to the vehicle group (control). One-way or two-way ANOVA followed by Tukey’s test, respectively, * *p* < 0.05 and ** *p* < 0.01 vs. control, *n* = 4–6 repetitions.

Concerning corticosterone levels (Figure 6), the repeated microdosing of *Pc*AE produced a significant reduction vs. the control group 7 days after the final administration (F_2,15_ = 4.25, *p* = 0.0345); in contrast, FLX only reflected a tendency to decrease these levels, (Figure 6A). Regarding the acute *Pc*AE macrodose, corticosterone concentrations were significantly lower compared to the control group at 24 h but not after 7 days of the last administration (treatment F_1,14_ = 3.87, *p* = 0.0691; time F_1,14_ = 8.48, *p* = 0.0114; interaction F_1,14_ = 7.21, *p* = 0.0948). Data revealed that corticosterone concentrations remained low 7 days post-administration within control mice (*p* = 0.0310) (Figure 6B).

## 3. Discussion

In this study, *P. cubensis* mushrooms were investigated with respect to preclinical anxiety-/depressive-like behavioral responses in mice, in addition to the reference drug FLX, and ECoG analysis was carried out on the parietal brain cortex and dendritic maturation of the hippocampus in the dentate gyrus area following acute and repeated microdosing (for 10 days) in comparison to a single macrodose to find evidence of the mushroom’s potential beneficial effects for mental disorders.

A reduction in indicators of depression and anxiety in patients suffering cancer and other mood disorders [24,25,26], as well as effects on addictive compounds [27,28], has been associated with treatment using an abundant alkaloid found in certain *Psilocybe* mushrooms, called psilocybin [29]. It is a chemical tryptamine alkaloid with a phosphoryl oxy substituent at the 4-position, for which its bioactive form, psilocin, is obtained after metabolism [30]. This information has prompted increased investigation of these alkaloids—psilocybin and psilocin—not only at macrodoses but also at microdoses. However, preclinical and clinical studies using a whole mushroom at microdoses or macrodoses are scarce, and there is still controversy regarding the clinical efficacy of microdosing with pure alkaloid compounds. A recent review highlighted a range of brain changes related to the microdosing of psychedelics in human trials, and it concluded that reduced doses are involved in neurobiological variations, states of consciousness, and augmented feelings of vigor and pain tolerance; however, there is currently insufficient evidence to determine whether these effects are therapeutic or placebo in nature [31].

In a preliminary study, the acute administration of *Pc*AE macrodoses exhibited antidepressant-like activity in a dose-dependent manner resembling that produced by FLX; these effects were significant from a dose of 1 mg/kg, i.p., of the extract, where psilocybin is involved as one of the bioactive metabolites, as its presence was confirmed by UPLC-MS analysis [14]. Dose–response effects have been explored following acute administration of the pure alkaloid psilocybin across a microdose range of 30 to 1000 µg/kg, as well as in time-course analysis using 100 to 1000 µg/kg in intracranial rat stimulation, suggesting therapeutic effects without potential abuse [32]. Psilocybin at 50–100 µg/kg, s.c., was also compared to the effects of ketamine at 300 µg/kg to 3 mg/kg, both reaching 7–12 ng/mL and 10–73 ng/mL in plasma samples of male Long-Evans rats, respectively, producing a modest change in the behavioral response of rats [33]. Nevertheless, *P. cubensis* mushrooms have not been sufficiently studied using preclinical or clinical microdosing. This investigation provides evidence, for the first time, of significant effects following acute and repeated administration of a microdose (1 µg/kg) of whole *P. cubensis* mushrooms, defined as 1/1000 of the effective dose of 1 mg/kg previously reported in mice [14]. These results suggest that different chemical constituents—other than alkaloids such as psilocybin detected in *Pc*AE—could act synergistically to produce central and even peripheral effects modulated by different mechanisms of action, as not only serotonergic but also noradrenergic action could be observed in the FST by an increase in swimming and climbing behaviors. Genomic and non-genomic actions are likely related to the anti-anxiety and antidepressant-like behavioral effects of *P. cubensis* mushrooms, as changes in neuronal activity were observed within the first 60 min after administration and persisted up to 24 h, promoting differential neuronal changes in the morphology of neurons.

Psilocybin at repeated low doses, s.c., has been associated with resilience to stress, where reduced frequencies of self-grooming suggest human compulsive behaviors [15]. Preclinical repeated treatment has also been investigated in the presence of psilocin at 50 and 75 µg/kg, administered three times over 6 days, to assess anxiolytic activity in the EPM using Wistar rats; modest effects equivalent to ketamine at 0.5 or 3 mg/kg were observed, with a mild anxiogenic response 48 h later [34]. In our study, repeated daily administration of an aqueous extract of *P. cubensis* at a microdose (1 µg/kg/day) for 10 days exhibited significant anxiolytic and antidepressant-like activity, similarly to the case of FLX (10 mg/kg/day). Repeated administration has already been reported only for FLX via daily treatment for 7 [35] or 14 days [36], with a significant reduction in behavioral responses to chronic variable or conditioned stress, respectively. In our study, the effects of repeated administration of *P. cubensis* did not exhibit any substantial differences compared to acute microdosing.

Regarding ECoG analysis, changes in frequency bands were dose-dependent between micro- and macrodoses, but no time-dependent effect between microdosing was observed in behavioral responses, as equivalent anxiolytic- and antidepressant-like behaviors after acute or repeated *Pc*AE microdosing (1 µg/kg, i.p.) were consistent with equivalent cortical activity in both schedules of administration. The activity was modest and selective, as only the alpha and beta frequency bands were modified. Interestingly, a dose-dependent effect was observed in ECoG activity, as significant changes were registered in the delta, alpha, and low-frequency bands (3–4 Hz) after the macrodose administration of *Pc*AE, in contrast to microdosing and the use of reference drug FLX. Similar data were reported for psilocybin (4 mg/kg), with a decrease in EEG registered in rats, and this decrease was more pronounced after 20–30 min and most evident between 50 and 60 min. A major EEG decrease in cortical areas was observed, such as the fronto–parieto–temporal cortices [37]. Psychedelic compounds are known to induce noticeable changes in cortical oscillatory activity, and they are characterized by broadband desynchronization [38] and large-scale network organization [39], which differs qualitatively from SSRI-induced modulation. Differences among microdoses and macrodoses suggest that their constituents and concentrations could be responsible for the effect of the mushroom, involving different mechanisms of action that require further studies. On the other hand, as human and animal behavior does not depend on a single brain center but rather on the coordinated and simultaneous activity of multiple brain regions and neural networks, the exploration of only one region may be a limitation of our study, as we are only reporting an analysis of the right parietal cortex of mice, which is associated with antianxiety and antidepressant behavioral responses. However, the data obtained from this cortex are relevant, as it is known that this area is involved in emotional regulation strategies through sensory integration/attention; its activation has been associated with anxiety, especially when faced with unpredictable threats. It is known that dysfunction may contribute to the cognitive impairment often seen in depression, such as deficits in spatial attention and working memory. It is also known that pharmacological modulation may therefore alter patterns of oscillatory coherence without necessarily producing proportional changes in behavioral phenotypes. Therefore, the apparent dissociation between ECoG activity and behavioral responses likely reflects distinct circuit-level mechanisms that ultimately converge on similar functional endpoints.

On the other hand, hippocampal neurogenesis comprises several processes, including stem cell recruitment and proliferation, maturation, neurite outgrowth, and synaptogenesis, among others. Decreased neurogenesis in the hippocampal area, mainly in the dentate gyrus, has been related to affective and psychiatric disorders, where pharmacological impact is produced by antidepressants and antipsychotics [40]. The antidepressant-like effects of FLX have been associated with increased survival and the facilitation of the neuronal maturation of newborn cells after chronic—but not acute—treatment, suggesting differential effects on hippocampal synaptic plasticity [41,42]. In our histological analysis and in agreement with these reports, early and significant neuronal changes were observed only after repeated administrations of FLX. Repeated administrations of *Pc*AE microdoses (1 µg/kg) exhibited a significant increase in category A cells, whereas category B and C cells were more evident in the FLX and post-macrodose treatment. Although dendritic maturation variations were observed after repeated *Pc*AE mushroom microdosing and macrodosing, behavioral responses were equivalent. This may be explained by differential control and synaptic mechanisms underlying the greater capacity of immature granular cells in the dentate gyrus to undergo synaptic potentiation. Increased excitability has been reported in newborn neurons, which exhibit a higher probability of generating action potentials compared with older cells in response to equivalent excitatory and inhibitory inputs [43]. This may be related to the early ECoG changes observed, with no differences between acute and repeated microdosing. To our knowledge, one recent investigation by Shahar et al. [44] described the effect of a macrodose of *Pc*AE mushrooms containing 1.3% psilocybin compared to synthetic psilocybin (4.4 mg/kg) in C57BL/6J male mice. A minimal increase in synaptic proteins was observed after three days of treatment, while by day 11, significant changes in synaptic protein expression (indicators of synaptic plasticity), such as GAP43, PSD95, synaptophysin, and SV2A, were detected in areas such as the prefrontal cortex, hippocampus, and limbic areas. Overall, the extract appeared to produce a greater and longer-lasting effect on synaptic plasticity than synthetic psilocybin [45]. These results reinforce the dose- and time-dependent effects of whole *P. cubensis* mushrooms, suggesting that not only psilocybin or psilocin alkaloids but also other compounds are likely involved—even in a synergistic interaction—in producing CNS effects, and they are mediated by non-genomic and genomic mechanisms of action.

Zheng et al. [46] report that the hippocampus and parietal cortex have multiple synapses between them that interact closely for effective navigation. This is a process that plays a fundamental role in cognition, allowing organisms to move through their environment more effectively. This interaction is relevant for neural mechanisms associated with navigation in situations involving rapid decision-making. According to our results, the anxiolytic- and antidepressant-like activities of the *Pc*AE treatment, which include exploration and motor coordination behavioral responses, modified electrical signals in the parietal cortex and promoted neural activity in the gyrus of the hippocampus mainly after macrodose administration, suggesting the important participation of these cerebral regions in the potential benefits of *Pc*AE in mental health.

It was a surprising finding to observe significant changes not only after repeated microdosing administration but also following a single administration of such a low dose. This dose was selected to demonstrate that a 1/1000 ratio of the *Pc*AE microdose (1 µg/kg, i.p.) would not be expected to alter behavioral responses in mice, whereas 1 mg/kg, i.p., does. Reduced changes in neuronal activity were observed in the ECoG, along with minimal induction of dendritic maturation changes, which increased significantly with macrodosing. These findings suggest that the CNS effects of this mushroom begin at this low microdose, indicating a wide dose range over which potential therapeutic effects of this *Psilocybe* mushroom may be observed in mental health contexts.

A limitation of this study is that the ECoG recording was performed in the parietal cortex, which prevents a complete assessment of network-level dynamics in the circuits involved in depressive and anxious behaviors. Future research will be required to understand functional connectivity through local field potential recordings in key corticolimbic and corticothalamic structures in order to better characterize the circuit-level effects of *P. cubensis* and its secondary metabolites. It is important to mention that the regimen and route of administration of the acute treatment of the reference drug FLX (three doses by subcutaneous injection) were taken from the literature to produce significant effects, but they were different to those used for *P. cubensis*, which might be a limitation of our experimental design, as the use of different routes of administration implies a pharmacokinetic process for obtaining final concentrations and response times. In addition, different injection number simply stress variations during the handling of mice, which could influence the effects between groups. Another limitation may be that no individual group of pure psilocybin was explored in this study due to its unavailability. However, our objective in this study was to understand the effect of the whole mushroom rather than the individual psilocybin compound, as its effects have already been reported in preclinical and clinical studies, and because people use *Psilocybe* mushrooms in folk medicine as a natural product. Finally, identifying the potential targets of this *Psilocybe* mushroom, not only for its anxiolytic/antidepressant effects but also those involved in neurogenesis or neuroplasticity, will reinforce its impact and utility for mental health.

## 4. Materials and Methods

### 4.1. Animals

A total of one hundred five male mice (Swiss Webster, weighing 25–30 g) (aged 8 ± 1 weeks old) were used in this study. Animals were provided by the INPRFM vivarium and maintained with free access to food and water at 22 ± 2 °C under a 12-h light/dark cycle. The experimental procedure followed the internationally accepted principles for laboratory animal use and care, as found in the European Community guidelines (2010/63/EU for the Protection of Laboratory Animals), the US guidelines (NIH publication #85-23, revised in 1985 and updated in 2011),and local guides such as the Norma Oficial Mexicana (NOM-062-ZOO-1999; México). The protocol was approved by the Animal Care and Use Committee of the INPRFM on 18 December 2024 (Approval No: CICUAL/02/2024). This project was approved by the following committees: Chemical Use Committee on 13 April 2023 (Approval No: Biosafety CB-005/2023) and Research Ethics Committee on 16 June 2025 (Approval No: CEI/C/022/2025).

### 4.2. Fungal Material

*P. cubensis* (Earle) Singer 1948 was collected in Xochicatlán, State of Hidalgo, Mexico (from June to September 2022). Dra. Leticia Romero Bautista, a biology, taxonomy, and mycology expert, identified this species. One specimen was sent to Escuela de Ciencias Biológicas, Instituto Politécnico Nacional, to be deposited at the Herbario “Gastón Guzmán Huerta” with reference No. 121805.

A chromatographic profile was first performed to identify psilocybin in a sample of 1 mg/mL of the extract dissolved in methanol (MeOH, HPLC-grade), followed by filtration through 0.22 µm filters (GHP, Acrodisc 13, Waters) in order to be injected into the chromatograph. The analysis was carried out using Empower chromatographic software version 3 (Waters, Milford, MA, USA) on a Waters Acquity UPLC H-Class liquid chromatography system fitted with a Waters photodiode array detector coupled with a mass spectrometer (UPLC-MS, Acquity Waters, Milford, MA, USA, (EE.UU.)). A Sorbax SB C-18 column (2.1 mm × 100 mm, 3.5 µm, Agilent, Santa Clara, CA, USA) with the thermostat set at 40 °C was used. The gradient system of the mobile phase consisted of Milli-Q water acidified with 0.1% acetic acid (solvent A) and HPLC-grade acetonitrile (solvent B). The analysis started with a gradient mixture of 70% A:30% B for a total time of 5 min using a constant flow rate of 0.3 mL/min; solvent B was progressively increased to reach 100%, and finally, it was returned to the original concentrations (70%:20%).

### 4.3. Drugs and Reagents

FLX (reference antidepressant drug) was purchased from Sigma (St. Louis, MO, USA) and dissolved in distilled water using subcutaneous (s.c., acute treatment) and intraperitoneal (i.p., repeated treatment) routes of administration. Isofluorane was acquired from Pisa Agropecuaria S.A. de C.V. (Mexico City; Mexico). The fungal material was resuspended in distilled water and administered via the parenteral and enteral (intraesophageal, p.o.) routes of administration. As i.p. injection provides rapid absorption, improved bioavailability, and faster distribution, it was selected to explore the effect of acute and repeated microdosing. Administration via i.p. was also considered for repeated administration in order to reduce discomfort in mice and the risk of esophageal irritation or damage. Drugs, reagents, and the aqueous extract were prepared close to the day of the experiment. The microdosing and macrodosing processes of *P. cubensis* mushrooms were chosen and prepared according to data from the literature and preliminary studies in our laboratory [14]. As the use of isotonic saline solutions can decrease the solubility of the extract components, causing precipitation, a decision was made to only use distilled water when administering *P. cubensis* mushrooms and the other treatments.

### 4.4. Experimental Design

**Figure 7:**
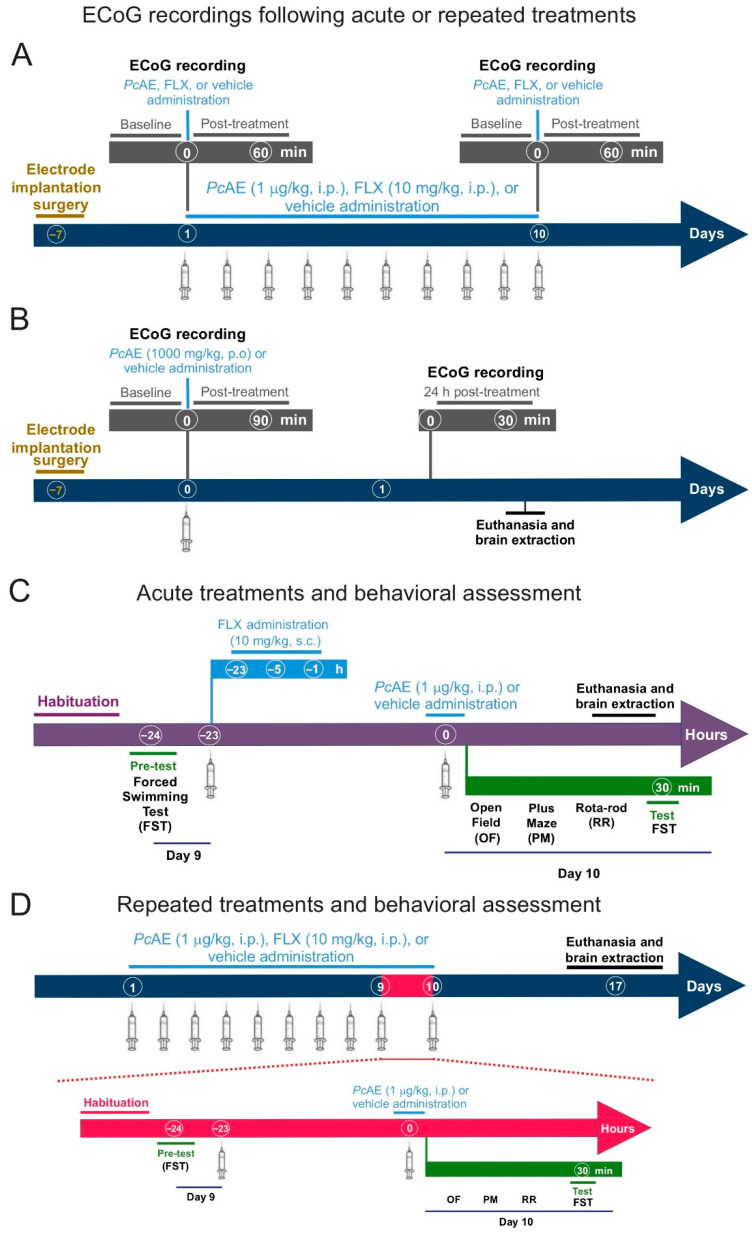
Timeline describing the experimental strategy of the electrocorticographic (ECoG) analysis after (**A**) acute or repeated microdosing of *P. cubensis* aqueous extract (*Pc*AE) and fluoxetine (FLX), as well as (**B**) acute *Pc*AE macrodosing. Panels (**C**,**D**) depict the behavioral assessment paradigms applied to evaluate anxiety-like behavior (open-field test, OF, before elevated plus-maze test, PM) and (**D**) depressive-like behavioral readouts (rota-rod test, RR, before the forced swimming test, FST) in mice. Brains were dissected after euthanasia for immunohistochemical analysis.

Fifty male mice, divided into groups of six to eight animals, were used to study the pharmacological activity of *P. cubensis.* Randomization was carried out using simple and block methods. The sample size was decided considering the use of the 3Rs principle, using the minimum number of animals required to achieve statistical significance and valid conclusions. An aqueous extraction (*Pc*AE) was investigated after acute or repeated microdosing (1 µg/kg, i.p.) administration (daily for 10 consecutive days) and acute and repeated FLX (10 mg/kg, s.c. and or i.p., respectively). Only an acute macrodose of 1000 mg/kg, p.o., in mice was administered (according to the Organization for Economic Co-operation & Development, major doses are considered for toxicological effects). Control mice received a vehicle (distilled water) or the clinical antidepressant drug FLX (10 mg/kg, s.c.). All treatments were carried out using 0.1 mL/10 g body weight. The reference drug was evaluated using three doses (23 h, 5 h, and 1 h before behavioral tests) for acute administration, as it is reported that a single dose does not produce significant effects [14] (see timeline in Figure 7). Because the assessments used in this study are short in duration, each mouse was identified with an individual number on the tail, marked using an alcohol-based felt marker. After randomization, mice were placed in boxes labeled with a key to blind the evaluation and subsequent data analyses. Different researchers were assigned to carry out these experiments, including personnel responsible for assigning, weighing, and identifying the mice; others performing the OF and/or rota-rod tests; others conducting the EPM; and others performing the FST. Behavioral responses were video-recorded for later evaluation, and other researchers were considered for data analysis.

Independent groups of mice were used to conduct the ECoG analysis exploring the acute or repeated microdosing of *Pc*AE (1 µg/kg, i.p.) or FLX (10 mg/kg, s.c. or i.p., respectively), and the effects were observed by recording electrical signals at baseline and at 30 to 60 min after treatment (Figure 7A).

In another experiment, ECoG analyses were investigated after macrodosing by recording data from the baseline and after 30 to 60 min of treatment. In this case, the time observation was extended to 90 min and 24 h after the treatment’s administration due to significant changes observed at 60 min in different frequency bands (Figure 7B).

Anxiolytic activity was evaluated in mice that received acute (Figure 7C) and repeated (Figure 7D) treatment in order to assay them in the elevated plus-maze test (EPM, 5 min with a preliminary OF exploration for 2 min), and then, the FST was carried out (previous analysis of their motor coordination in the rota-rod). Each apparatus in all tests was cleaned with a disinfecting solution (70% ethanol) after evaluating each mouse. Behavioral responses to the antidepressant-like effect of treatments were estimated 30 min after the last administration. For the FST evaluation, the water in the tank was changed each time a new evaluation was performed on each mouse.

After 7 days of behavioral testing, euthanasia by decapitation was considered to collect the blood from the trunk in order to determine corticosterone concentrations, while brains were processed for doublecortin (DCX) immunohistochemistry. Since significant effects of *Pc*AE macrodoses on ECoG activity were observed at 24 h, the brains were not only subsequently analyzed at 7 days but also 24 h after administration (Figure 7).

### 4.5. Stereotaxic Surgery and Electrocorticographic (ECoG) Recording

For the in situ ECoG, fifty-five mice were first anesthetized under isoflurane for a stereotaxic implantation of three electrodes: two in the parietal cerebral cortices and one over the cerebellum [47]. Postoperative recovery of 7 days was allowed for mice for the experimental ECoG analysis. Two independent experiments were included to estimate the effects of *Pc*AE on ECoG activity: The first was assessed in thirty-six animals divided into six groups of six mice to evaluate the effects of acute and repeated microdosing, while the second focused on macrodosing using twelve mice divided into two groups of six animals. Electrode implantation was performed using a Stoelting stereotactic frame (Stoelting, Wood Dale, IL, USA). Electrodes were positioned in the parietal cortex at the coordinates relative to the bregma (AP−3 mm; ML 2.5 mm), and an additional reference electrode was placed over the cerebellum (AP−5 mm; ML 0), according to the mouse brain atlas [46], to target the parietal cortical region involved in cortico-limbic processing; this method was previously used in electrophysiological studies assessing antidepressant-related activity.

During microdosing, the same group of mice was recorded after receiving an acute injection of *Pc*AE (1 µg/kg, i.p.) and on day 10 after daily administration to evaluate the effect of acute and repeated treatments. FLX (10 mg/kg, i.p., reference drug) was evaluated simultaneously. Each recording consisted of a 30 min baseline and 60 min post-treatment, divided into two periods of 30 min (Figure 7A). Similarly, for the macrodosing experiment, ECoG recordings began 30 min before treatment to establish baseline activity, but in this case, the analysis was followed by 90 min and 24 h post-treatment (Figure 7B).

The ECoG signals were acquired from the parietal cortices using the GRASS Model 8-18D polygraph with a bandwidth of 1–70 Hz, digitized at a sampling rate of 500 Hz; the spectral analysis was performed using custom routines developed in MATLAB (2016a MATLAB, The Mathworks Inc., Natick, MA, USA), employing the built-in *fft* function [48]. Power spectra were obtained in the 1–50 Hz broadband from five 60s sections corresponding to the reference line 25–30 min, 55–60 min, 90 min, and 24 h post-treatment. Absolute power values were normalized from 0 to 1 and separated into seven frequency bands as follows: delta (1–4 Hz), theta (4–8 Hz), alpha (8–13 Hz), beta (13–30 Hz), gamma (30–50 Hz), and an additional 3–6 Hz band [14,49,50]. Seven mice were excluded because they exhibited artifacts during the ECoG recording or lost their recording connector during the experiment; these animals were euthanized as a humane endpoint.

### 4.6. Behavioral Evaluation

#### 4.6.1. Open-Field Test (OF)

To rule out motor impairments that could affect behavioral performance in the EPM and FST, the OF test was performed. The test consisted of an opaque Plexiglas box (40 × 30 × 20 cm) divided into12 squares (6 × 6 cm),with each mouse placed in a corner. The ambulatory activity was determined by registering the quadrangles explored within 2 min [51]. Rearing behavior was measured in mice by counting the frequency with which mice stood on their hind extremities and supported both forelimbs against the acrylic box during a 2-min period as an indicator of the depressant effect. The first two minutes was considered, as mice exhibit the highest exploratory and rearing behavior during this period.

#### 4.6.2. Elevated Plus-Maze Test (EPM)

The EPM consisted of a wooden cross elevated 50 cm from a base, with two open arms (30 × 5 cm) and two closed ones (30 × 15 × 5 cm) and a central open space (5 × 5 cm) in which each mouse was placed. This test confirmed the anxiolytic activity of the treatments. The parameters used to determine the anxiolytic effect in both closed and open arms were registered as previously reported within a period of 5 min [14,52,53].

#### 4.6.3. Rota-Rod Test

The motor coordination ability of mice was assessed on a Rota-Rod Treadmill (Rota-Rod Treadmills for mice, constant-speed model 7600, Ugo Basile; 4 cm diameter). The protocol consisted of two stages. (1) Training and Selection: Mice were tested for their balancing ability on a rotating bar at a constant speed of 16 rpm for 2 min. Animals that demonstrated successful motor performance in this stage were included for group randomization. Under this criterion, the inclusion rate exceeded 80%; excluded animals were reassigned to different experimental protocols. (2) Experimental Stage: Thirty minutes after treatment administration, mice were placed again on the rota-rod for a 2-min session before the swimming test. Since the rota-rod test is performed on mice for 2 min prior to the forced swim test (FST), the possibility that conducting the tests in this sequence may have influenced the FST results due to factors such as stress or fatigue in the mice cannot be ruled out. Therefore, the group receiving the vehicle is crucial, as it allows comparison with the group treated with doses of the whole *P. cubensis* mushroom, as previously reported [14].

#### 4.6.4. Modified Forced Swimming Test (FST)

After 24 h, a swimming (15 min) pre-test was carried out in a transparent glass cylinder (26 cm height × 12 cm diameter) filled with 17 cm of tap water (25 ± 2 °C). Experiments were performed between 9 and 13 h with illumination at 18 lux intensity. The animals underwent a final 5-min session in which behavioral responses were scored based on immobility (minimal movement used to keep the head above water or floating), swimming (slight movements around the glass cylinder), and climbing (vigorous movements against the walls of the cylinder) [54]. A time sampling technique was used to record the number of counts every 5 s for a total duration of 5 min [55]. At the end of the test, mice were removed, towel-dried, and placed in a recovery area with sawdust under a red-light lamp for 15 min before being returned to their home cages. All sessions were videotaped and analyzed by an observer blinded to the experimental groups.

#### 4.6.5. Immunohistochemistry

The mouse brain was processed for immunohistochemistry using 40 µm coronal slices obtained from a complete hippocampus (AP: −1.34 to −3.80 from bregma) via microtome equipment (Leica, Buffalo Grove, IL, USA) [56]. Slices were deposited in a cryoprotectant liquid at 4 °C and were kept there until staining was carried out using the immunohistochemistry process [55].

To visualize doublecortin (DCX) expression, sections were processed using free-floating immunohistochemistry with a peroxidase-based method in every sixth tissue slice of the dentate gyrus, separated by 240 µm [57]. DCX-labeled cells were recognized using primary DCX antibodies (1:100; Abcam, Cambridge, MA, USA) and secondary biotinylated anti-rabbit antibodies (1:2:50; Jackson ImmunoResearch, West Grove, PA, USA). The density and dendritic labeling of DCX-positive cells were analyzed every 200 µm throughout the granular cell layer of the hippocampus [58]. Tissue sections were viewed under a light microscope to capture photographs with a camera (Leica, Buffalo Grove, IL, USA), and the free ImageJ software (version 1.53) for Windows was used.

#### 4.6.6. Determination of Morphology Category

Neuronal density and complexity were determined based on the morphological characteristics of DCX-positive cells [59]. Three stages were defined according to soma size and dendritic morphology: (A) proliferative cells, comprising DCX-labelled cells with absent or smaller dendrites; (B) intermediate cells, with larger and longer dendrites than those in category A; and (C) post-mitotic cells, characterized by a single primary dendrite larger than those in intermediate category, often with other nodes, indicative of more complex newborn neurons [60].

#### 4.6.7. Quantification of Corticosterone via the Enzyme-Linked ImmunoSorbent Assay (ELISA) Method

After decapitation, corticosterone levels were measured following the instructions of the ELISA kit (Enzo Life Sciences, Farmingdale, NY, USA) using serum obtained from mouse blood samples (centrifuged at 15,000 rpm at 4 °C for 15 min). Microplate absorbance was read at 405 nm using an ELISA reader (Bio-Tek, Shoreline, Washington, DC, USA).

### 4.7. Statistical Analysis

Data from ECoG recordings, behavioral responses, and neuronal morphology are expressed as mean ± standard error of the mean (S.E.M.) from at least 6 replicates. Statistical analysis was performed using one or two-way analysis of variance (ANOVA) for significant differences, followed by Tukey’s or Sidak’s post hoc tests depending on the repeated measures or two-group comparisons or if the comparison was vs. the control group or between factors, such as treatment (A) and time (B). Student’s *t*-test was considered in specific cases when two groups were compared: for example, macrodosing vs. the control. GraphPad Prism software (2019) Version 8.0.2 for Windows was used to validate data, and *p* < 0.05 was considered significant.

## 5. Conclusions

In conclusion, our present study complements preliminary preclinical scientific evidence on the anxiolytic and antidepressant-like properties of *Psilocybe* mushrooms associated with a wide range of doses, highlighting a dose- and time-dependent effect in the brain to promote CNS depressant neuronal activity and possible neuroplasticity. Our findings, taken together, emphasize the interest of this natural resource for mental disorder therapy.

## Data Availability

The presented data in this study are available upon request.

## Abbreviations

The following abbreviations are used in this manuscript:

ANOVA	Analysis of Variance
AP	Anteroposterior
CNS	Central Nervous System
DCX	Doublecortin
ECoG	Electrocorticographic
FLX	Fluoxetine
ELISA	Enzyme-Linked ImmunoSorbent Assay
FST	Forced Swimming Test
GHP	Glutathione Hydroxy Peroxidase
H	Hours
Hz	Hertz
HPLC	High-Performance Liquid Chromatography
INPRFM	Instituto Nacional de Psiquiatría Ramón de la Fuente Muñiz
i.p.	Intraperitoneal
kg	Kilogram
MDD	Major Depressive Disorder
Min	Minutes
nm	Nanometer
MeOH	Methanol
mg	Milligram
OF	Open Field Test
*Pc*AE	*Psilocybe cubensis* Aqueous Extract
*P. cubensis*	*Psilocybe cubensis*
EPM	Elevated Plus Maze Test
RR	Rota-Rod
p.o.	Intragastric Administration
SSRIs/SNSIs	Selective Serotonin and/or Norepinephrine Reuptake Inhibitors
SARS-CoV-2	Severe Acute Respiratory Syndrome Coronavirus2
SEM TRD	Standard Error of the Mean Treatment-Resistant Depression
UPLC H-Class	Ultra-Performance Liquid Chromatography H-Class
UPLC-MS	Ultra-Performance Liquid Chromatography–Mass Spectrometry
UV-MS	Ultraviolet Spectroscopy and Mass Spectrometry
WHO	World Health Organization
µg	Microgram
µm	Micrometer
ng	Nanogram
GAP43	Growth-Associated Protein 4
PSD95	Postsynaptic Density Protein 9
SV2A	Synaptic Vesicle Glycoprotein 2A
SECIHTI	Secretaría de Ciencia, Humanidades, Tecnología e Innovación
CONAHCYT	Consejo Nacional de Humanidades, Ciencias y Tecnologías
